# An essay on the Charcot and Richer hysteria: from charcoal drawings to cell phones

**DOI:** 10.1055/s-0044-1789229

**Published:** 2024-08-31

**Authors:** Marlon Wycliff Caeira, Leo Coutinho, Igor Abrahim Nascimento, Luciano de Paola, Hélio Afonso Ghizoni Teive

**Affiliations:** 1 Universidade Federal do Paraná, Hospital de Clínicas, Serviço de Epilepsia, Curitiba PR, Brazil.; 2 Universidade Federal do Paraná, Hospital de Clínicas, Serviço de Distúrbios do Movimento, Curitiba PR, Brazil.; 3 Universidade Federal do Paraná, Departamento de Medicina Interna, Programa de Pós-Graduação em Medicina Interna, Curitiba PR, Brazil.

**Keywords:** Hysteria, Conversion Disorder, Dissociative Disorders, Epilepsy, Epilepsy, Tonic-Clonic, Histeria, Transtorno Conversivo, Transtornos Dissociativos, Epilepsia, Epilepsia Tônico-Clônica

## Abstract

Hysteria, previously also known as the disease of the womb, has moved from being a woman's illness through the medieval times' stigma of demonic possession, to the modern concept of a functional neurological disorder. Interestingly to the present assay, Charcot (1825–1893) and Richer (1849–1933) described, in their 1887 work *Les Démoniaques dans l'art* , by means of iconography, semiological aspects of the so-called *Grande Attaque Hystérique* , which resembles features of psychogenic nonepileptic seizures emulating grand mal epileptic seizures. The aim of the present assay is to describe how those charcoal iconographic representations evolved through history and are nowadays portrayed in videos recorded at epilepsy monitoring units and patients' cell phones.

## Resumo

Histeria, previamente também conhecida como a doença do útero, passou de uma doença feminina, pelo estigma de possessão demoníaca ao longo dos tempos medievais, até o conceito moderno de um distúrbio neurológico funcional. Curiosamente para o presente ensaio, Charcot (1825–1893) e Richer (1849–1933) descreveram, em sua obra *Les Démoniaques dans l'art* , de 1887, por meio da iconografia, aspectos semiológicos do chamado *Grande Attaque Hystérique* , que se assemelha às características de crises não epilépticas psicogênicas que emulam crises epilépticas do tipo grande mal. O objetivo deste ensaio é descrever como essas representações iconográficas evoluíram ao longo da história e são retratadas nos dias de hoje em vídeos gravados em unidades de monitoramento de epilepsia e nos celulares de pacientes.

## INTRODUCTION

Hysteria, or the suffocation of the mother, 1 2 as described by Edward Jorden (1569–1632), has metamorphosed from a disease specific to women and their wombs (from the Greek *hysterikós* , “relative to the womb”) to the concept of functional neurologic disorders. 3 In fact, Jorden's considerations on hysteria figure among the firsts attempts to demystify its medieval misconception, as a work of witchcraft and manifestation of demonic possession in the female body, heading back to the Hippocratic argument of a genuine disease whose pathology relies on the connections of the womb to many body systems and whose symptoms were “monstrous and terrible to behold.” 2 4 After Jorden, many eminent physicians, such as Thomas Sydenham (1624–1689) and, later, Philippe Pinel (1745–1826), also defined hysteria as an illness, either organic or mental. 4 5 In addition to recognizing it as an emulator of almost all organic ailments, Sydenham also stated for the first time that this malady is not restricted to women, also affecting men of “sedentary or studious lives,” removing the uterus from the main stage and presenting the brain as candidate for its origin. 4 5 This last theory was also shared by his contemporaries Charles Lepois (1563–1633) and Thomas Willis (1621–1675), as well as by Pierre Briquet (1796–1881), over a century later. 6 7

Jean-Martin Charcot (1825–1893) stands out in the history of hysteria both scientifically and by means of spectacle and art, due to his “Leçons du Mardi à la Salpêtrière,” brilliantly documented by his disciples Bourneville (1840–1909) and Regnard (1850–1927) in the repository *Iconographie Photographique de la Salpêtrière* , and his work with Paul Richer (1849–1933), *Les Démoniaques dans l'art* 6 8 . Of interest to this assay is the first part of the latter opus, which describes *les démoniaques convulsionnaires* (the convulsive demons) or *La Grande Attaque Hystérique* (the great hysterical attack), with Richer's personal drawings to represent the so-called hysteroepileptic phenomena resembling *grand mal* seizures and their four periods, as previously proposed by Charcot: *période épileptoide* (epileptoide period), *grands mouvements/clownisme* (great movements/clownism), *attitudes passionnelles* (emotional gestures), and *Période terminale* (final delirium). 8 9 In the book's second part, Charcot recognizes a variation of the third phase of the attack, which he calls *les extatiques* (the ecstatic), which could include quietness, feelings of ecstasy, and negative sensory phenomena such as blindness, delusional speech, and hallucinations, often with religious or even erotic connotations, referencing many masterpieces of religious art, which has been reviewed elsewhere. 10

Charcot's saints are a beautiful and visionary attempt to describe psychogenic nonepileptic seizures (PNES) or functional seizures and other stereotyped neurological events in relation to the “sacred disease” (that is, epilepsy) and its uttermost presentation: grand mal seizures. The aim of the present work is to make a brief reference to PNES as represented by Charcot in his use of the iconography of saints and how those same signs are perceived under the lenses of modern epilepsy monitoring units (EMUs), as a puzzle piece on the often difficult diagnosis of PNES.

## METHODS

A comparison between Charcot and Richer's iconography in *Les Démoniaques dans l'arts* and our institution's repository of video-electroencephalograms (vEEGs) recorded between 1996 and 2019 was performed to find typical characteristics of PNES also ascribed in Charcot's times to the *grande attaque hystérique* and its semiological phenomena. All patients consented to the use of their data for academic purposes.

## RESULTS

**Figure 1:**
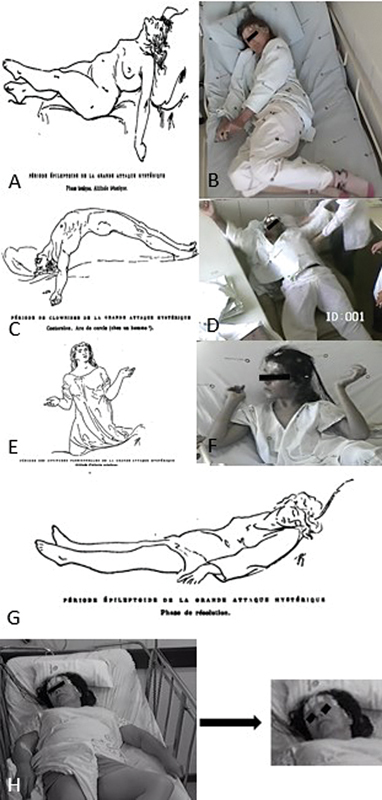
( **A** ) *Période épileptoide* of the hysterical attack from *Les Démoniaques dans l'art* . 8 ( **B** ) Similar semiological features are present in a patient in our practice diagnosed with psychogenic non-epileptic seizure (PNES), who shows both upper limbs in extension, crossed legs, tilted head, and closed eyes and fists, along with unresponsiveness during the functional seizure. ( **C** ) *Période de clownisme* and *grands mouvements* in a male patient in the Richer drawing depicted with opisthotonus − the *arc de cercle* ( **D** ) and in a patient of ours diagnosed with PNES. ( **E** ) *L'attitude passionnelle* , the contemplative attitude found in *Les Extatiques* ( **F** ) and the same facial expression in a patient of ours with the diagnosis of PNES after vEEG. ( **G** ) *Période épileptoide, phase de résolution* − after the seizure-like phenomena of the *Période épileptoide* , follows the resolution phase of the hysteric attack as depicted by Richer. ( **H** ) A similar asthenic expression was also observed in one of our patients following a functional seizure. The patients' eyes were blacked out to preserve their identities for ethical purposes.

**Figure 2:**
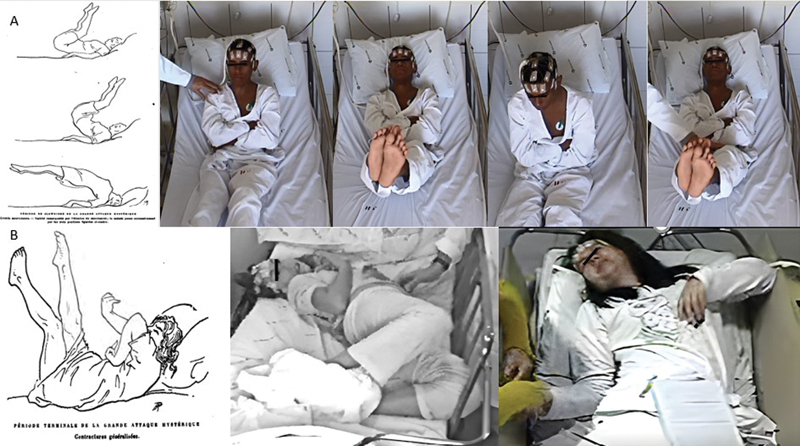
( **A** ) *Grands mouvements* . Pelvic thrusting movements and sustained elevation of the legs present in the clownism phase of the *grande attaque hystérique* as depicted in *Les Démoniaques dans l'arts* . 8 On the frames of the upper panel, a young male patient presents alternating movements of the lower limbs and trunk over the hips, both arms were crossed in a posture unfit for an epileptic seizure. The movements could be stopped and be induced by the touch of the technician. ( **B** ) In the lower panel, one can see the generalized contractures in the *période terminale* of the attack as depicted by Charcot and Richer, and examples of generalized contractures and bizarre postures at the end of a functional seizure. Both patients tend to opisthotonus, with the patient on the left lying down with both eyes open and a similar dystonic posture of the hand as shown in the drawing, while the patient at the botton right of the figure presents with hand drop, eyes closed and an asymmetrical contracture of head and neck muscles with jaw deviation to the left. The patients' eyes were blacked out to preserve their identities for ethical purposes.

Frames from 7 vEEGs were selected and are shown in Figures 1 , 2 , representing the four canonical phases of the *grande attaque hystérique* , along with the proper remarks.

## DISCUSSION

The International League Against Epilepsy (ILAE) defines PNES as paroxysmal, time-limited, alterations with motor, sensory, autonomic, and/or cognitive signs, as well as symptoms not caused by ictal epileptiform activity. 11 They represent approximately 10% of seizures in the emergency room, encompassing from 5 to 10% of patients under care for epilepsy, and 20 to 40% of the diagnosis in tertiary epilepsy centers. 12 13 More importantly, between 50 and 81% of all PNES seizures will emulate grand mal seizures 14 and, in spite of their often bizarre presentation, they are commonly mistaken by epileptic seizures, delaying the correct diagnosis by a mean of 7 years, increasing treatment cost and morbidity. 15

Over the past 40 years, based on systematic analysis of vEEGs, several clinical discriminators between epileptic seizures and PNES have been proposed. 11 15 Remarkable examples include the ictal eye closure, opisthotonus, and hand clawing. 15 16

Despite the fact that sensorial symptoms (that is, the Charcot stigmata) may be considered an archetypical manifestation of hysteria, these phenomena may also be found in epileptic seizures (such as focal onset non-motor seizures). But, when present in PNES, they commonly pose anatomical incompatibilities with motor symptoms. 17 On a similar account, the ecstatic crisis can also be related to epilepsy, often related to the nondominant temporal lobe. 10 18

Regardless their accuracy, the indisputable fact is that these stigmata of PNES were reported by a set of remarkable clinicians unaided by technology and subjected to the mystical and religious influence of their time. Nonetheless, their observations survived through the sieve of time, and remain as solid and inspirational clinical tools, influencing the diagnostic skills of young physicians armed with home videos sent to their cell phones. It is worth mentioning the work by Amin et al., 19 who investigated 44 patients from their epilepsy center and found 94% of agreement between the interpretations of two blinded physicians for the ictal phenomena in question – either epileptic or not – when comparing the patients' standard vEEGs to smartphone homemade videos. There are other reports based on vEEG, highlighting pictorial traits in drawings as useful clinical hints to teach how to discriminate between epileptic and nonepileptic phenomena. 17

Stepping away from the epileptology, it is important to briefly address the social and anthropological ramifications of Charcot's works. Being an anticlerical thinker, he spent his life advocating, although less actively than his pupil Bourneville, for the secularization of medical science. His work in *Les Démoniaques dans l'art* is a firm expression of this belief, defying the dominant religious ideas of nineteenth century Parisian society. 6 20

Charcot's privileged clinical mind and Richer's fine tracing combined shed an initial light on the challenging field currently known as neurological functional disorders. At the end of the day, the accuracy and detailing of their observations own very little to the findings on our videos today, even without the freezing, framing, and rewinding capabilities.
